# Durability of bioprosthetic aortic valve replacement in patients under the age of 60 years — 1-year follow-up from the prospective INDURE registry

**DOI:** 10.1093/icvts/ivad115

**Published:** 2023-07-18

**Authors:** Bart Meuris, Jean-Christian Roussel, Michael A Borger, Matthias Siepe, Pierluigi Stefano, Günther Laufer, Thierry Langanay, Alexis Theron, Martin Grabenwöger, Konrad Binder, Philippe Demers, Renzo Pessotto, Wouter van Leeuwen, Thierry Bourguignon, Sergio Canovas, Giovanni Mariscalco, Enrico Coscioni, Francois Dagenais, Olaf Wendler, Gianluca Polvani, Matthias Eden, Beate Botta, Peter Bramlage, Ruggero De Paulis

**Affiliations:** Cardiac Surgery, University Hospitals Leuven, Leuven, Belgium; Service de chirurgie thoracique et cardiovasculaire, CHU Nantes, Nantes, France; Department for Cardiac Surgery, Leipzig Heart Center, University of Leipzig, Leipzig, Germany; Department of Cardiovascular Surgery, University Heart Center, University Hospital Freiburg, Freiburg, Germany; Faculty of Medicine, Albert-Ludwigs-University of Freiburg, Freiburg, Germany; Department of Cardiac Surgery, University Hospital Bern, University of Bern, Switzerland; Division of Cardiac Surgery, Careggi University Hospital, Florence, Italy; Department of Cardiac Surgery, Medical University of Vienna, Vienna, Austria; Thoracic and Cardiovascular Surgery, Rennes University Hospital Center, Rennes, France; Cardio-Thoracic Surgery Department, Hospital de la Timone, Marseille, France; Department of Cardiovascular Surgery, HZH Heart Center Hietzing, Austria; Heart Center University St. Pölten, St. Pölten, Austria; Department of surgery, Montreal Heart Institute, University of Montreal, Montreal, Canada; Edinburgh Heart Centre, Royal Infirmary of Edinburgh, Edinburgh, UK; Cardio-Thoracic Surgery, Erasmus University Medical Center, Rotterdam, Netherlands; Department of Cardiology and Cardiac Surgery, Tours University Hospital, Tours, France; Cardiovascular Surgery Department, Hospital University Virgen de la Arrixaca, Murcia, Spain; National Institute for Health Research Leicester Biomedical Research Centre, Glenfield Hospital, Leicester, England; Division of Cardiac Surgery, University Hospital San Giovanni di Dio e Ruggi d’Aragona, Salerno, Italy; Institute University, Cardiology Center, Québec, Canada; Department of Cardiothoracic Surgery, King’s College Hospital NHS Foundation Trust, London, UK; Department of Cardiovascular Surgery, Cardiology Center Monzino, Milan, Italy; Department for Internal Medicine III, Molecular Cardiology and Angiology, University Hospital Schleswig-Holstein, Kiel, Germany; Institute for Pharmacology and Preventive Medicine, Cloppenburg, Germany; Institute for Pharmacology and Preventive Medicine, Cloppenburg, Germany; 25. Department of Cardiac Surgery, European Hospital, Rome, Italy

**Keywords:** Surgical aortic valve replacement, Structural valve degeneration, Valve durability

## Abstract

**OBJECTIVES:**

We report 1-year safety and clinical outcomes in patients <60 years undergoing bioprosthetic surgical aortic valve intervention.

**METHODS:**

The INSPIRIS RESILIA Durability Registry is a prospective, multicentre registry to assess clinical outcomes of patients <60 years. Patients with planned SAVR with or without concomitant replacement of the ascending aorta and/or coronary bypass surgery were included. Time-related valve safety, haemodynamic performance and quality of life (QoL) at 1 year were assessed.

**RESULTS:**

A total of 421 patients were documented with a mean age of 53.5 years, 76.5% being male and 27.2% in NYHA class III/IV. Outcomes within 30 days included cardiovascular-related mortality (0.7%), time-related valve safety (VARC-2; 5.8%), thromboembolic events (1.7%), valve-related life-threatening bleeding (VARC-2; 4.3%) and permanent pacemaker implantation (3.8%). QoL was significantly increased at 6 months and sustained at 1 year. Freedom from all-cause mortality at 1 year was 98.3% (95% confidence interval 97.1; 99.6) and 81.8% were NYHA I versus 21.9% at baseline. No patient developed structural valve deterioration stage 3 (VARC-3). The mean aortic pressure gradient was 12.6 mmHg at 1 year and the effective orifice area was 1.9 cm^2^.

**CONCLUSIONS:**

The 1-year data from the INSPIRIS RESILIA valve demonstrate good safety and excellent haemodynamic performance as well as an early QoL improvement.

**Clinical trial registration:**

clinicaltrials.gov: NCT03666741.

**Figure:**
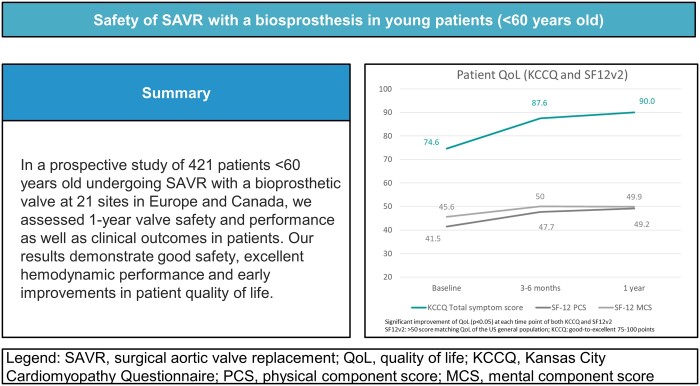

## INTRODUCTION

While mechanical valves have traditionally been preferred over bioprosthetic valves in younger patients, the use of bioprosthetic valves has expanded due to their durability, decreased risk of reoperation and the possibility of undergoing a transcatheter valve-in-valve procedure [1]. Retrospective observational studies have reported comparable long-term benefits in patients 50–69 years undergoing mechanical versus bioprosthetic valve replacement [2, 3]. As a result, current American and European guidelines recommend lower age cut-offs (50–65 years of age) for the use of bioprostheses, emphasizing the importance of considering individual patient factors and informed shared decision-making [4, 5].

The INSPIRIS RESILIA aortic valve (AV) (Edwards Lifesciences, Irvine, CA, USA) is a stented bioprosthetic, tri-leaflet valve comprised of bovine pericardial tissue. To date, 1 pre-clinical randomized controlled trial [6] and several clinical trials [7–12] involving the RESILIA tissue were performed. Flameng *et al.* [6] reported significantly improved haemodynamic and anticalcification properties of the RESILIA tissue compared with the standard Perimount valve in the juvenile sheep model. The findings from a single-arm registry and the COMMENCE trial have shown excellent safety and effectiveness at 5 years, with no structural valve deterioration (SVD) [7, 11]. The INSPIRIS RESILIA valve has also demonstrated improved haemodynamic performance in early results of smaller cohorts [10, 12].

Although data on safety and effectiveness of the RESILIA tissue are accumulating, studies focusing specifically on younger patients <60 years are lacking. The prospective INSPIRIS RESILIA Durability Registry (INDURE) aims to provide data on short-term clinical effectiveness, as well as on long-term haemodynamic and structural performance in patients <60 years. Here, we report 1-year data of patients enrolled.

## METHODS

INDURE is a prospective, open-label, multicentre, international registry to assess the clinical outcomes of patients younger than 60 years of age who undergo surgical aortic valve replacement (SAVR) with the INSPIRIS RESILIA AV [13]. Patients were enrolled at 21 sites across Austria, Belgium, France, Germany, Italy, Netherlands, Spain, the UK and Canada.

### Ethics statement

The ethics committee responsible for each site granted approval, and written informed consent was obtained.

### Patients

Adult patients 60 years of age or younger, undergoing SAVR and receiving the INSPIRIS RESILIA AV prosthesis were enrolled. In addition to the stipulations of the device Instructions for Use, inclusion criteria included a planned replacement of the native valve as indicated based on a preoperative evaluation. The aortic valve replacement (AVR) was either isolated or with concomitant replacement of the ascending aorta and/or coronary artery bypass graft. Patients undergoing pulmonary vein isolation were also allowed if it was not a full cox-maze procedure. Patients with (i) no possibility of valve implantation in accordance with the Instructions for Use; (ii) presence of active or within the last 3 months of the scheduled SAVR endocarditis/myocarditis; (iii) previous AVR; (iv) a Bentall (root) procedure or any surgery on other valves; or (v) life expectancy of <12 months were excluded.

### Objectives

The primary objective was to determine the time-related valve safety at 1 year depicted as freedom from events in patients undergoing SAVR and receiving the INSPIRIS RESILIA AV prosthesis. Time-related valve safety was defined as composite end point according to the valve academic research consortium (VARC)-2 criteria [requiring of repeat procedure; prosthetic valve endocarditis, prosthetic valve thrombosis, thromboembolic events (e.g. stroke) and life-threatening bleeding] [14]; however, due to more precise definitions compared to VARC-2, SVD stage 3 was presented according to VARC-3 criteria comparing 1 year versus discharge echo [increase in mean AV pressure gradient (PG) ≥20 mmHg resulting in mean AV PG ≥30 mmHg with a concomitant decrease in effective orifice area (EOA) ≥0.6 cm^2^ or ≥50% and/or a decrease in doppler velocity index (DVI) ≥0.2 or ≥40%, OR new occurrence, or increase of ≥2 grades, of intraprosthetic AR resulting in severe AR] [15].

The secondary objective was the assessment of haemodynamic performance of the INSPIRIS RESILIA AV and further durability parameters, clinical outcomes and quality of life (QoL). Further clinical outcomes of interest were all-cause, cardiovascular and valve-related mortality [15], valve-related dysfunction, requirement of repeat procedure due to any cause, permanent pacemaker implantation, Acute Kidney Injury Network stage 2/3 and New York Heart Association (NYHA) functional class compared to baseline. QoL was assessed using the Kansas City Cardiomyopathy Questionnaire (KCCQ) and Short Form-12 Health Survey Version 2 (SF-12v2).

Outcomes according to the VARC-2 criteria were adjudicated by an independent clinical event committee. Digital imaging and communication in medicine files of echocardiograms generated at 1-year follow-up were collected for analysis by the Echo Core Laboratory to ensure unbiased and consistent analysis of the diagnostic data.

### Statistical analysis

Data were analysed using descriptive statistics, with categorical variables presented as absolute values and frequencies (%) and the continuous variables presented as means [standard deviation (SD)] and/or median [interquartile range (IQR)]. Test for normal distribution was carried using the Kolmogorov–Smirnov test. Wilcoxon signed ranks test for paired data was used for comparing QoL scores between baseline and follow-up visits. For outcome reporting Kaplan–Meier estimates were provided. A *P*-value of <0.05 was considered statistically significant. Statistical analysis was performed using SPSS Version 28.0 (Armonk, NY, IBM Corp.) [16].

## RESULTS

**Figure 1:**
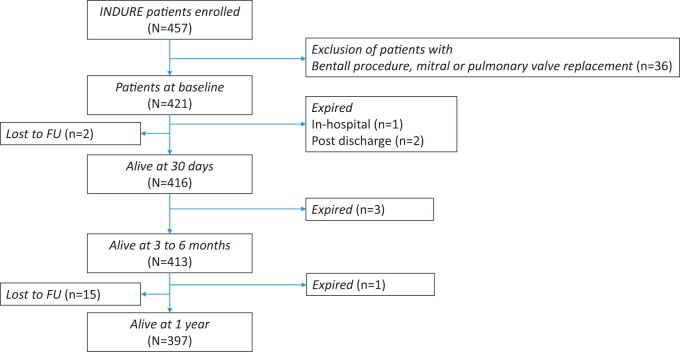
Study flowchart. FU: follow-up.

A total of 457 patients were enrolled between April 2019 and May 2021. For the present analysis, 36 patients with a Bentall procedure and mitral/pulmonary valve replacement were excluded, resulting in a total of 421 patients. Within the first-year post-SAVR, 17 patients were lost to follow-up (4.0%). Of the remaining 404 patients, 7 (1.7%) patients died, which resulted in a total of 397 (94.3%) patients alive with available data at 1 year (Fig. 1).

### Patient characteristics

Patients had a mean age of 53.5 (SD: 6.9) years [median 55 (IQR: 51, 58)], primarily male (76.5%) with a mean body mass index (BMI) of 28.2 (SD: 5.1) kg/m^2^. 27.2% had NYHA class III/IV and 5.3% had Angina Canadian Cardiovascular Society (CCS) III/IV (Table 1). The mean European System for Cardiac Operative Risk Estimation II was 1.5% [SD: 1.6%; median 0.95% (IQR: 0.67, 1.83)] and Society of Thorasic Surgeons (STS) risk score 1.1% [SD: 1.0%; median 0.75% (IQR: 0.50, 1.20)]. Common comorbidities included arterial hypertension, coronary artery disease and type II diabetes. The mean left ventricular ejection fraction was 59.3%, the EOA index was 0.54 cm^2^/m^2^, the mean AV PG was 45.3 (SD: 21.5) mmHg and left ventricular outflow tract diameter was 23.8 (SD: 10.6) mm. In patients with pure aortic stenosis (AS), the mean aortic PG at 1 year was 52.9 (SD: 16.3) mmHg and the EOA index was 0.41 (SD: 0.14) cm^2^/m^2^.

**Table 1:** Patient characteristics

	Total population
	Mean (SD) or *n* (%)
Age (years)	53.5 (SD: 6.9)
Female gender	99 (23.5)
Body mass index (kg/m^2^)	28.2 (SD: 5.1)
NYHA class III/IV	113 (27.2)
Angina CCS class III/IV	22 (5.3)
EuroScore II (%)	1.5 (SD: 1.6)
	0.95 (IQR: 0.67, 1.83)
STS risk score (%)	1.06 (SD: 0.99)
	0.75 (IQR: 0.50, 1.20)
Medical history	
Coronary artery disease	99 (23.5)
Hypertension	209 (49.6)
Prior MI	16 (3.8)
Prior PPI	12 (2.9)
Prior PCI	24 (5.7)
Diabetes	55 (13.1)
Peripheral vascular disease	16 (3.8)
Prior stroke/TIA	24 (5.7)
COPD	32 (7.6)
Renal failure (eGFR >50)/dialysis	5 (1.2)
Echocardiographic variables	
Severe AV stenosis	294 (70.0)
Severe AV regurgitation	92 (21.9)
LVEF (%)	59.3 (SD: 10.1)
EOA (cm^2^)	1.07 (SD: 0.76)
EOA index (cm^2^/m^2^)	0.54 (SD: 0.39)
Peak AV pressure gradient (mmHg)	70.6 (SD: 33.3)
Mean AV pressure gradient (mmHg)	45.3 (SD: 21.5)
Vmax (m/s)	4.0 (SD: 1.1)
Severe pulmonary hypertension, >55 (mmHg)	6 (1.6)

AV: aortic valve; COPD: chronic obstructive pulmonary disease; EuroScore: European System for Cardiac Operative Risk Estimation; EOA: effective orifice area; eGFR: glomerular filtration rate; LVEF: left ventricular ejection fraction; MI:myocardial infarction; PCI: percutaneous coronary intervention; PPI: permanent pacemaker implantation; SD: standard deviation; TIA: transient ischaemic attack

### Procedural details

The prevalence of bicuspid valves was 73.2% (Table 2). A total of 346 (82.4%) patients in the overall population had AS of any severity and 277 (66.0%) had AR. AS was dominating in 304 (72.4%) patients while AR was dominating in 98 (23.3%) patients. Pure forms of AS and AR were present in 142 (33.8%) and 73 (17.4%) patients. The aetiology of valve pathology in the overall population was as follows: 73.6% were congenital, 22.8% were degenerative, 1.0% were rheumatic, 0.7% were endocarditic and 1.9% (*n* = 8) were other/unknown (*n* = 5 unknown, *n* = 2 prolaps/pocket rupture and *n* = 1 dilation of aortic root).

**Table 2:** Interventional details and discharge

	Mean (SD), median (IQR) or *n* (%)
Bicuspid valve	308 (73.2)
Pure stenosis	142 (33.8)
Pure regurgitation	73 (17.4)
Mixed disease (stenosis and regurgitation)	205 (48.8)
Aetiology of valve pathology	
Degenerative	96 (22.8)
Congenital	310 (73.6)
Rheumatic	4 (1.0)
Endocarditic	3 (0.7)
Other^a^/unknown	8 (1.9)
Surgical approach	
Full sternotomy	302 (71.7)
Upper hemisternotomy	112 (26.6)
Right anterior mini thoracotomy	7 (1.7)
Isolated aortic valve replacement	255 (60.6)
Concomitant procedure	
Coronary artery bypass graft	53 (12.6)
1 graft	23
2 grafts	19
3 grafts	11
Root replacement	6 (1.4)
Supracoronary tube graft	78 (18.5)
Other	59 (14.0)
Implantation details	
First attempt successful^b^	417 (99.0)
Paravalvular leakage (visible)	7 (1.7)
Second attempt needed	
Successful	4
2nd cross-clamp	2
Valve size Edwards INSPIRIS	25 (IQR: 23, 25)
Intraoperative complication	
Aortic rupture/dissection	3 (0.7)
Coronary artery obstruction	1 (0.2)
Conversion to full sternotomy	2 (0.5)
Duration of intervention	
Procedure time (min)	197 (SD: 59) 186 (IQR: 155, 230)
Cross clamp time (min)	74.2 (SD: 25.2) 70 (IQR: 55, 88)
Cardiopulmonary bypass (min)	96.4 (SD: 33.8) 89 (IQR: 72, 116)
Length of stay	
Hospital stay (implant to discharge) (days)	8.4 (SD: 4.3) 7 (IQR: 6, 10)
Intensive care unit length of stay (h)	50.4 (SD: 55.9) 30 (IQR: 22, 56)
Duration of mechanical ventilation (h)	10.1 (SD: 31.0) 6 (IQR: 4, 9)

a Prolaps/pocket rupture (*n* = 2), aortic root dilation (*n* = 1).

b Aortic rupture/dissection (*n* = 1), coronary artery obstruction (*n* = 1), multiple complications (*n* = 1) and paravalvular leakage (*n* = 1).

IQR: interquartile range; SD: standard deviation.

**Figure 2:**
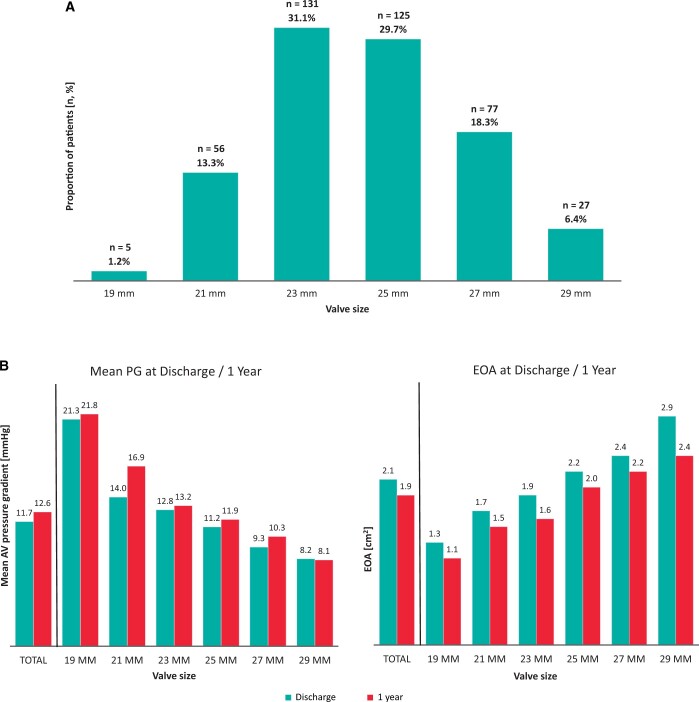
(**A**) Valve size distribution and (**B**) haemodynamics over time by valve size. AV: atrioventricular; EOA: effective orifice area; PG: pressure gradient.

The common surgical approach was full sternotomy (71.7%), followed by upper hemisternotomy (26.6%) and right anterior minithoracotomy (1.7%) (Table 2). Isolated AVR was performed in 255 (60.6%) patients, of whom 163 (63.4%) received full sternotomy. In the total cohort, the median valve size was 25 mm as the majority of patients received either a 23-mm valve (31.1%) or a 25-mm valve (29.7%) (Fig. 2A). A total of 5 (1.2%) 19-mm valve were implanted with all patients being female. Intraoperative complications were as follows: 3 (0.7%) patients had aortic rupture or dissection, 2 (0.5%) patients required conversion to full sternotomy and 1 (0.2%) patient had coronary artery obstruction. There were no cases of intraoperative death.

### Procedural and in-hospital outcomes

Patients stayed for 8.4 (SD: 4.3) days in the hospital and 50.4 (SD: 55.9) h in the ICU. The mean duration of mechanical ventilation was 10.1 (SD: 31.0) h (Table 2). There was a decrease in mean aortic PG and an increase in EOA depending on valve size: patients receiving a 19-mm valve had the highest mean aortic PG at discharge (21.3 mmHg) and smallest EOA (1.3 cm^2^), while those receiving a 29-mm valve had lowest mean aortic PG (8.2 mmHg) and largest EOA (2.9 cm^2^) (Fig. 2B). Severe patient–prosthesis mismatch was present in 4 (1.0%) patients. One (0.2%) patient died in the hospital, over a half of the patients were discharged home [*n* = 245 (58.0%)] and 156 (37.1%) were referred to cardiac rehabilitation.

**Figure 3:**
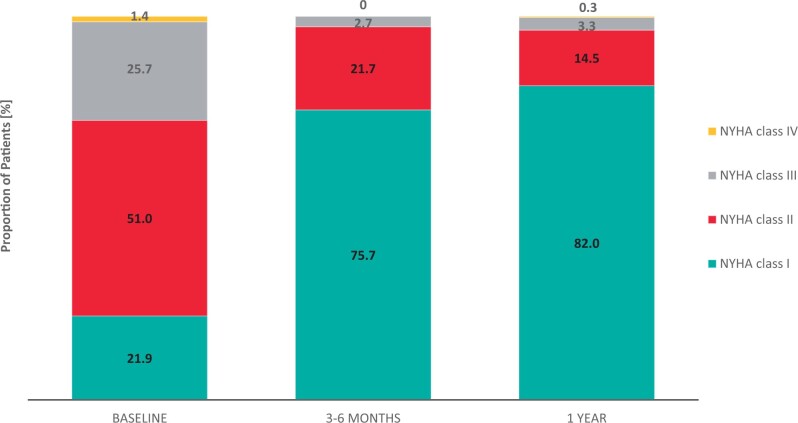
NYHA functional class versus baseline. NYHA: New York Heart Association.

Compared to discharge, mean aortic PG was only slightly higher at 1 year while EOA was lower in patients (Fig. 2B). In addition, 81.8% of patients were in NYHA class I at 1 year compared to 21.9% at baseline and only 3.6% were in NYHA class III/IV compared to 27.2% at baseline (Fig. 3). There were no cases of mild/severe PVL at 1 year.

### Quality of life outcomes

**Figure 4:**
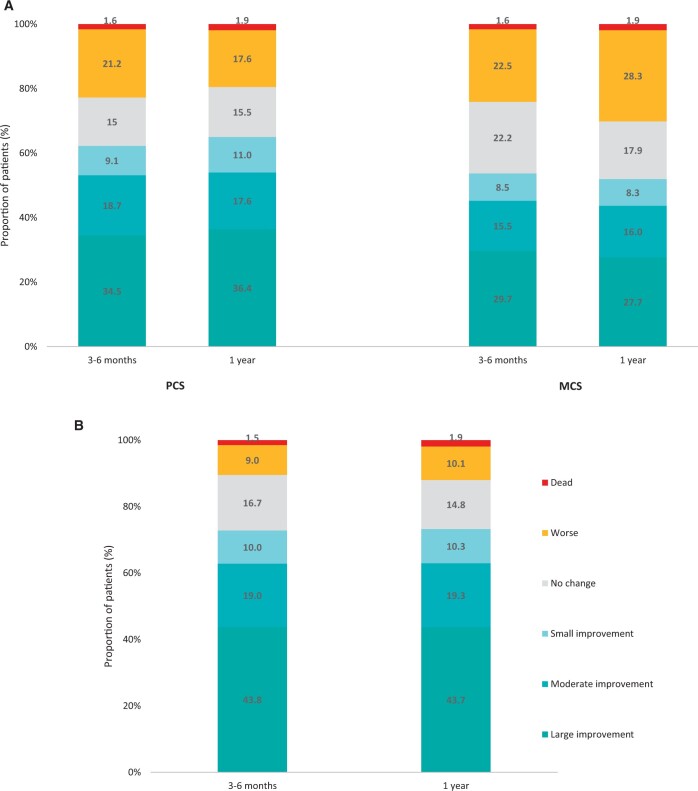
Quality of life changes versus baseline. (**A**) SF-12v2 and (**B**) KCCQ. KCCQ: Kansas City Cardiomyopathy Questionnaire; MCS: mental component summary; PCS: physical component summary; SF-12v2: Short Form-12 Health Survey Version 2.

SF-12v2 and KCCQ were used for assessing QoL in patients (Table 3 and Supplementary Material, Table S1). The mean SF-12v2 physical summary score at 3–6 months was 47.7 points (*P* < 0.001) compared to baseline (41.5 points) with a further increase at 1 year (49.2 points; *P* < 0.001). The mean SF-12v2 mental summary score at 3–6 months was 50.0 points (*P*  < 0.001) from baseline (45.6 points) and remained relatively stable at 1 year (49.9 points; *P*  < 0.001). Both physical and mental summary scores at 1 year were near the general population mean (50.0 points). Overall, changes in PCS and MCS at 1 year compared to baseline were classified as follows: 39.6% and 29.8% of patients had a large improvement in QoL. 1.9% for both died (Fig. 4).

**Table 3:** Quality of life

	Baseline	3–6 months		1 year	
	(*N* = 397)	(*N* = 394)	(*N* = 378)	(*N* = 380)	(*N* = 365)
	Mean (SD)	Mean (SD)	*P*-Value* (versus baseline)	Mean (SD)	*P*-Value* (versus baseline)
SF-12v2					
Physical component summary	41.5 (SD: 10.5)	47.7 (SD: 9.7)	<0.001	49.2 (SD: 9.5)	<0.001
Mental component summary	45.6 (SD: 11.2)	50.0 (SD: 10.5)	<0.001	49.9 (SD: 10.6)	<0.001
KCCQ	(*N* = 401)	(*N* = 399)	(*N* = 384)	(*N* = 385)	(*N* = 371)
Total symptom score	74.6 (SD: 22.6)	87.6 (SD: 17.2)	<0.001	90.0 (SD: 17.1)	<0.001
Overall summary score	66.1 (SD: 22.7)	85.2 (SD: 17.5)	<0.001	87.1 (SD: 18.0)	<0.001
Clinical summary score	75.1 (SD: 21.2)	88.4 (SD: 15.6)	<0.001	89.2 (SD: 16.4)	<0.001

* Based on paired cases.

KCCQ: Kansas City Cardiomyopathy Questionnaire; SD: standard deviation; SF-12v2: Short Form-12 Health Survey Version 2.

The KCCQ total symptom score and clinical summary score significantly increased both at 3–6 months (87.6 points; *P*  < 0.001 and 88.4 points; *P*  < 0.001) and at 1 year (90.0 points; *P*  < 0.001 and 89.2 points; *P*  < 0.001) compared to baseline (74.6 and 75.1 points) (Table 3). Similarly, there was an increase in mean overall summary score already at 3–6 months (85.2 points; *P*  < 0.001) and further at 1 year (87.1 points; *P*  < 0.001) in comparison to baseline (66.1 points). Overall, 1-year changes in patient overall summary score from baseline were classified as follows: 43.7% of patients had a large improvement in QoL and 1.9% died (Fig. 4).

### Outcome events at 30 days and 1 year

At 30 days, a total of 3 (0.7%) patients had died, with all cases being due to cardiovascular reasons: (Table 4). At 1 year, further 4 [1.0%/valve years (vy)] patients died (total *n* = 7 at 1 year): 2 (0.5%/vy) deaths were due to cardiovascular causes and 2 (0.5%/vy) were related to non-cardiovascular factors. There were no cases of valve-related death. Freedom from all-cause mortality at 6 months and 1 year was 98.8% [95% confidence interval (CI) 97.8; 99.8] and 98.3% (95% CI 97.1; 99.6). Freedom from valve-related mortality was 100% at all timepoints.

**Table 4:** Early and late outcomes

	Early (≤30 days), *n* (%)	Late (>30 days to 1 year), *n* (%/vy)^a^	Freedom from event (6 months), % (95% CI)	Freedom from event (1 year), % (95% CI)
All-cause mortality	3 (0.7)	4 (1.0)	98.8 (97.8, 99.8)	98.3 (97.1, 99.6)
Cardiovascular	3 (0.7)	2 (0.5)	99.3 (98.5, 100.0)	98.8 (97.7, 99.8)
Valve related^b^	0 (0)	0 (0)	100.0	100.0
Time-related valve safety (VARC-2)	25 (5.9)	9 (2.2)	93.1 (90.6, 95.5)	91.8 (89.1, 94.4)
SVD stage 3 (VARC-3)^c^	–	0 (0)	–	100.0
Prosthetic valve endocarditis	0 (0)	1 (0.2)	99.8 (99.3, 100.0)	99.8 (99.3, 100.0)
Prosthetic valve thrombosis	0 (0)	4 (1.0)	99.5 (98.9, 100.0)	99.0 (98.1, 100.0)
Thromboembolic event	7 (1.7)	5 (1.2)	98.1 (96.8, 99.4)	97.1 (95.4, 98.7)
Stroke	3 (0.7)	0 (0)	99.3 (98.5, 100.0)	99.3 (98.5, 100.0)
Valve-related bleeding				
Life threatening	18 (4.3)	0 (0)	95.7 (93.8, 97.7)	95.7 (93.8, 97.7)
Other outcomes				
Valve-related dysfunction	1 (0.2)	4 (1.0)	99.8 (99.3, 100.0)	98.7 (97.6, 99.8)
Requirement of repeat procedure (all cause)^d^	0 (0)	1 (0.2)	99.8 (99.3, 100.0)	99.8 (99.3, 100.0)
Valve-related bleeding (total)^e^	45 (10.7)	0 (0)	89.3 (86.4, 92.3)	89.3 (86.4, 92.3)
Permanent pacemaker implantation	16 (3.8)	3 (0.7)	95.7 (93.7, 97.6)	95.4 (93.4, 97.4)
Acute kidney injury AKIN stage 2/3	6 (1.4)	0 (0)	98.6 (97.4, 99.7)	98.6 (97.4, 99.7)

a 406 valve years.

b Within the first year, it was unknown in a total of 4 patients whether the death was valve related.

c SVD stage 3 according to VARC-3 comparing 1 year versus discharge echo (increase in mean AV PG ≥20 mmHg resulting in mean AV PG ≥30 mmHg with a concomitant decrease in EOA ≥0.6 cm^2^ or ≥50% and/or a decrease in doppler velocity index (DVI) ≥0.2 or ≥40%, OR new occurrence, or increase of ≥2 grades, of intraprosthetic aortic regurgitation (AR) resulting in severe AR).

d Requiring repeat procedure of the prosthetic valve due to prosthetic endocarditis.

e Valve-related bleeding reported as minor, major and life threatening according to VARC-2.

AKIN: Acute Kidney Injury Network; AV: aortic valve; CI: confidence interval; EOA: effective orifice area; PG: pressure gradient; SVD: structural valve deterioration; vy: valve years.

Time-related valve safety events (VARC-2) were reported in 25 (5.9%) patients as early (≤30 days) and 9 (2.2%/vy) patients as late outcome (>30 days to 1 year) with freedom from event of 91.8% (95% CI 89.1; 94.4) at 1 year. None of the patients developed SVD stage 3 according to VARC-3 criteria within the first-year post-AVR. No patient developed prosthetic valve endocarditis and valve thrombosis as early outcomes, but at 1 year, the incidence was 1 (0.2%/vy) and 4 (1.0%/vy), respectively. Two of the patients with valve thrombosis, the patient with endocarditis and 2 further patients [mild PVL post-AVR due to severe annular calcification (*n* = 1) and new onset of mild AV regurgitation (*n* = 1)] developed valve-related dysfunction at 1 year (total *n* = 5); re-AVR was needed solely in the patient with endocarditis. Thromboembolic events were documented in 7 (1.7%) patients as early outcome [of whom 3 (0.7%) were strokes], and in 5 (1.2%/vy) patients as late outcome. Valve-related life-threatening bleeding according to VARC-2 occurred in 18 (4.3%) patients as early outcome (mainly being revision for bleeding) with no incidence at 1 year.

Total valve-related bleeding (categorized in minor, major and life threatening) occurred in 45 (10.7%) patients as early outcome and there were no further cases at 1 year. Sixteen (3.8%) patients required a permanent pacemaker implantation as early outcome and further 3 (0.7%/vy) patients required it as late outcome. Six (1.4%) patients developed Acute Kidney Injury Network stage 2/3 at 30 days with no further incidence at 1 year.

## DISCUSSION

The 1-year results of the INDURE demonstrate (i) high hospital and 1-year survival rates with an absence of valve-related mortality; (ii) satisfactory and stable performance of the INSPIRIS RESILIA with complete freedom from stage 3 SVD based on a standardized CoreLab adjudicated assessment; and (iii) an improvement in the patients’ QoL early after the intervention which was sustained at 1 year.

### Hospital and 1-year survival rates

In-hospital all-cause mortality rate in our study (0.7%) was lower than the rates reported in the trials by Useini (2.5%) and Fukunaga (3.4%), which both evaluated hospital outcomes after AVR using the INSPIRIS RESILIA bioprosthesis in smaller cohorts [10, 12]. The reported in-hospital mortality rates for the Carpentier-Edwards Perimount Magna Ease bioprosthesis with RESILIA tissue range between 1.2% and 2.3% [7, 8].

We report excellent survival rates at 1 year: overall survival was 98.3% and valve-related survival was 100%. Although survival may vary depending on patient characteristics, the survival rates in our patient cohort are comparable or potentially even slightly better to those reported in previous trials using bioprostheses with RESILIA tissue [7, 8]. Puskas *et al.* [7] reported a 1-year overall survival of 97.6% and valve-related survival of 98.8% with the Carpentier-Edwards Perimount Magna Ease bioprosthesis (Model 11000A) in the COMMENCE trial. Bartus *et al.* [9] reported an overall mortality rate of 6.8% for the same bioprosthesis. Furthermore, Didier *et al.* [17] reported higher mortality rates after transcatheter aortic valve replacement (TAVR) with balloon-expandable transcatheter heart valves at 1 year (23.2%).

### Performance of the INSPIRIS RESILIA

One-year haemodynamic performance of INSPIRIS RESILIA, evaluated by an independent CoreLab, was favourable. Mean aortic PG [12.5 (SD: 5.3) mmHg] and EOA [1.9 (SD: 0.6) cm^2^] at 1 year were within the ranges reported in other studies. In the COMMENCE trial, the mean aortic PG and EOA at 1 year were 10.4 (SD: 4.9) mmHg and 1.7 (SD: 0.5) cm^2^ [7]. Bartuś *et al.* reported mean aortic PG and EOA at 1 year to be 13.9 (SD: 6.1) mmHg and 1.8 (SD: 0.6) cm^2^ [8]. The mean aortic PG and EOA reported in a Japanese cohort undergoing AVR with INSPIRIS RESILIA were 11.2 (SD: 3.2) mmHg and 1.8 (SD: 0.4) cm^2^. In the recently published early results after INSPIRIS RESILIA AVR (including only discharge data), the mean aortic PG was 10.2 (SD: 4.1) mmHg, which is slightly lower than the mean aortic PG at discharge in our cohort [11.7 (SD: 4.3) mmHg]. It should be noted, however, that patients receiving 19-mm valves (*n* = 5; all female) in our study exhibited elevated mean aortic PG at both discharge (21.3 mmHg) and 1 year (21.8 mmHg). Increased aortic PG may lead to risks associated with patient–prosthesis mismatch as well as accelerated degeneration of the implanted valve. Therefore, it is important to provide reduced gradients to patients requiring smaller valves, particularly those requiring a 19-mm valve.

It is known that the use of bioprosthetic valves is associated with higher rates of SVD, particularly in younger patients. Although high freedom from SVD at 1 year in the current study further highlights the durability of RESILIA tissue reported in previous studies [7–9], it is important to note that the rates of SVD in the first years are generally very low and the incidence rises in later years. However, SVD is caused by degenerative calcification over time in the majority of cases, which can be permanently reduced by the novel integrity preservation technology applied during the preparation of RESILIA tissue [6]. This preservation technology is described as a capping process, which permanently blocks residual aldehyde content known to bind with calcium. Further glycerolization preserves the tissue in dry storage, which provides a persistent protection of collagen.

The rates of prosthetic valve endocarditis and prosthetic valve thrombosis at 1 year were 0.2% and 1.0%, respectively. A recent meta-analysis concluded that bioprosthetic valves may be associated with a higher risk of endocarditis than mechanical valves [18]. However, freedom from prosthetic valve endocarditis in our patient cohort was still very high (99.8%) and comparable to the rates previously reported for RESILIA tissue [7–9]. The prevalence of prosthetic valve thrombosis in our study (1.0%) is comparable with that reported in the literature (0.6–0.7%), although the authors state that their prevalence is currently underestimated since routine prospective follow-up imaging is frequently not performed in the absence of symptoms or haemodynamic changes noted by echocardiography [19]. It has also been reported that the risk of thrombosis is higher with stented bioprosthetis, such as INSPIRIS RESILIA, compared to stentless valves [20]. In addition, we did not differentiate between clinical valve thrombosis and subclinical leaflet thrombosis characterized by hypoattenuated leaflet thickening, which is defined as an incidental finding of an increase in the thickness of the prosthetic valve leaflets without associated symptoms. Hypoattenuated leaflet thickening may be an early indicator of valve thrombosis, although its relationship to clinical events is still not clear [21, 22]. Therefore, we feel that the prevalence of prosthetic valve thrombosis after valve implantation in our study is acceptable.

### Quality of life

We assessed QoL in patients in this study, which has not been reported in previous trials on valves with the RESILIA tissue [7–9]. Myken *et al.* assessed the differences between patients receiving mechanical and bioprosthetic valves for heart valve surgery and found no differences [23]. Repack *et al.* [24] also compared postoperative QoL in patients undergoing aortic root replacement with mechanical versus bioprosthetic valves and reported similar outcomes in QoL between 2 patient groups. In our study, there was a significant improvement in QoL already at 6 months post-surgery, with further improvement at 1 year, suggesting that SAVR with INSPIRIS RESILIA improves QoL in young patients.

### Limitations

The INDURE provides real-world data of a large patient cohort with the applicability of findings to clinical practice across Europe and Canada. However, as we did not include an active control group, different bioprosthetic valves or valve generations could not be compared and selection bias cannot be excluded. Furthermore, there is no comparison of the bioprosthetic valve data with the outcomes and performance of mechanical valves. Lastly, although the results presented here are limited to 1-year data and may not reflect the ultimate safety outcomes and performance of the valve prosthesis, the present cohort will be followed up for 5 years, indicating the reporting of long-term outcomes in the future.

## CONCLUSION

The results of this study showed good safety outcomes, early improved QoL and high survival at 1 year in patients under the age of 60 receiving the INSPIRIS RESILIA valve.

## Supplementary Material

ivad115_Supplementary_Data

## Data Availability

All relevant data within this article will be shared upon reasonable request to the corresponding author.

## ABBREVIATIONS

AV: Aortic valve
CI: Confidence interval
EOA: Effective orifice area
INDURE: INSPIRIS RESILIA Durability Registry
IQR: Interquartile range
KCCQ: Kansas City Cardiomyopathy Questionnaire
PG: Pressure gradient
QoL: Quality of life
SD: Standard deviation
SF-12v2: Short Form-12 Health Survey Version 2
SVD: Structural valve deterioration
vy: Valve years

## References

[1] Christ T , Grubitzsch H, Claus B, Konertz W. Stentless aortic valve replacement in the young patient: long-term results. J Cardiothorac Surg 2013;8:68. 23566631 10.1186/1749-8090-8-68 PMC3639088

[2] McClure RS , McGurk S, Cevasco M, Maloney A, Gosev I, Wiegerinck EM et al Late outcomes comparison of nonelderly patients with stented bioprosthetic and mechanical valves in the aortic position: a propensity-matched analysis. J Thorac Cardiovasc Surg 2014;148:1931–9. 24521965 10.1016/j.jtcvs.2013.12.042

[3] Chiang YP , Chikwe J, Moskowitz AJ, Itagaki S, Adams DH, Egorova NN. Survival and long-term outcomes following bioprosthetic vs mechanical aortic valve replacement in patients aged 50 to 69 years. JAMA 2014;312:1323–9. 25268439 10.1001/jama.2014.12679

[4] Vahanian A , Beyersdorf F, Praz F, Milojevic M, Baldus S, Bauersachs J et al; ESC/EACTS Scientific Document Group. 2021 ESC/EACTS Guidelines for the management of valvular heart disease. Eur Heart J 2022;43:561–632. 34453165 10.1093/eurheartj/ehab395

[5] Writing Committee M , Otto CM, Nishimura RA, Bonow RO, Carabello BA, Erwin JP 3rd, et al ACC/AHA Guideline for the management of patients with valvular heart disease: a report of the American College of Cardiology/American Heart Association Joint Committee on Clinical Practice Guidelines. J Am Coll Cardiol 2020;77:e25–e197. 33342586 10.1016/j.jacc.2020.11.018

[6] Flameng W , Hermans H, Verbeken E, Meuris B. A randomized assessment of an advanced tissue preservation technology in the juvenile sheep model. J Thorac Cardiovasc Surg 2015;149:340–5. 25439467 10.1016/j.jtcvs.2014.09.062

[7] Puskas JD , Bavaria JE, Svensson LG, Blackstone EH, Griffith B, Gammie JS et al; COMMENCE Trial Investigators. The COMMENCE trial: 2-year outcomes with an aortic bioprosthesis with RESILIA tissue. Eur J Cardiothorac Surg 2017;52:432–9. 28605428 10.1093/ejcts/ezx158

[8] Bartuś K , Litwinowicz R, Kuśmierczyk M, Bilewska A, Bochenek M, Stąpór M et al Primary safety and effectiveness feasibility study after surgical aortic valve replacement with a new generation bioprosthesis: one-year outcomes. Kardiol Pol 2018;76:618–24. 29297188 10.5603/KP.a2017.0262

[9] Bartus K , Litwinowicz R, Bilewska A, Stapor M, Bochenek M, Rozanski J et al Intermediate-term outcomes after aortic valve replacement with a novel RESILIA(TM) tissue bioprosthesis. J Thorac Dis 2019;11:3039–46. 31463133 10.21037/jtd.2019.07.33 PMC6688015

[10] Useini D , Schlomicher M, Haldenwang P, Naraghi H, Moustafine V, Bechtel M et al Early results after aortic valve replacement using last generation bioprosthetic aortic valve. Heart Surg Forum 2021;24:E598–E962. 10.1532/hsf.4189 34962472

[11] Bavaria JE , Griffith B, Heimansohn DA, Rozanski J, Johnston DR, Bartus K et al; COMMENCE Trial Investigators. Five-year outcomes of the COMMENCE trial investigating aortic valve replacement with RESILIA tissue. Ann Thorac Surg 2023;115:1429–36. 35065065 10.1016/j.athoracsur.2021.12.058

[12] Fukunaga N , Yoshida S, Shimoji A, Maeda T, Mori O, Yoshizawa K et al Hemodynamic performance of INSPIRIS RESILIA aortic bioprosthesis for severe aortic stenosis: 2-year follow-up in Japanese cohort. J Artif Organs 2022;25:323–8. 35129732 10.1007/s10047-022-01316-5

[13] Meuris B , Borger MA, Bourguignon T, Siepe M, Grabenwoger M, Laufer G et al Durability of bioprosthetic aortic valves in patients under the age of 60 years - rationale and design of the international INDURE registry. J Cardiothorac Surg 2020;15:119. 32460798 10.1186/s13019-020-01155-6 PMC7251702

[14] Kappetein AP , Head SJ, Genereux P, Piazza N, van Mieghem NM, Blackstone EH et al; Valve Academic Research Consortium (VARC)-2. Updated standardized endpoint definitions for transcatheter aortic valve implantation: the Valve Academic Research Consortium-2 consensus document (VARC-2). Eur J Cardiothorac Surg 2012;42:S45–60. 23026738 10.1093/ejcts/ezs533

[15] Généreux P , Piazza N, Alu MC, Nazif T, Hahn RT, Pibarot P et al; VARC-3 WRITING COMMITTEE. Valve Academic Research Consortium 3: updated endpoint definitions for aortic valve clinical research. Eur Heart J 2021;42:1825–57. 33871579 10.1093/eurheartj/ehaa799

[16] IBM SPSS Statistics for Windows, Version 28.0. Armonk, NY: IBM Corp, 2021

[17] Didier R , Eltchaninoff H, Donzeau-Gouge P, Chevreul K, Fajadet J, Leprince P et al Five-year clinical outcome and valve durability after transcatheter aortic valve replacement in high-risk patients. Circulation 2018;138:2597–607. 30571260 10.1161/CIRCULATIONAHA.118.036866

[18] Anantha-Narayanan M , Reddy YNV, Sundaram V, Murad MH, Erwin PJ, Baddour LM et al Endocarditis risk with bioprosthetic and mechanical valves: systematic review and meta-analysis. Heart 2020;106:1413–9. 32471905 10.1136/heartjnl-2020-316718

[19] Pournazari P , Chang SM, Little SH, Goel S, Faza NN. Prosthetic aortic valve thrombosis. US Cardiol Rev 2022;16:e17 2022. 10.15420/usc.2021.19 PMC11588168 39600844

[20] Dangas GD , Weitz JI, Giustino G, Makkar R, Mehran R. Prosthetic heart valve thrombosis. J Am Coll Cardiol 2016;68:2670–89. 27978952 10.1016/j.jacc.2016.09.958

[21] Blanke P , Leipsic JA, Popma JJ, Yakubov SJ, Deeb GM, Gada H et al; Evolut Low Risk LTI Substudy Investigators. Bioprosthetic aortic valve leaflet thickening in the evolut low risk sub-study. J Am Coll Cardiol 2020;75:2430–42. 32234463 10.1016/j.jacc.2020.03.022

[22] Halperin JL , Zagha D. When Should We Go With HALT? JACC Cardiovasc Imaging. 2016 Dec 8:S1936-878X(16)30893-2. doi: 10.1016/j.jcmg.2016.11.002. 10.1016/j.jcmg.2016.11.002 28017713

[23] Myken P , Larsson P, Larsson S, Berggren H, Caidahl K. Similar quality of life after heart valve replacement with mechanical or bioprosthetic valves. J Heart Valve Dis 1995;4:339–45. 7582138

[24] Repack A , Ziganshin BA, Elefteriades JA, Mukherjee SK. Comparison of quality of life perceived by patients with bioprosthetic versus mechanical valves after composite aortic root replacement. Cardiology 2016;133:3–9. 26389590 10.1159/000438783

