# Supplementary material for: Durability of bioprosthetic aortic valve replacement in patients under the age of 60 years — 1-year follow-up from the prospective INDURE registry
Source: Interdiscip Cardiovasc Thorac Surg. 2023 Jul 18;37(4):ivad115. doi: 10.1093/icvts/ivad115 (PMC10576637; doi:10.1093/icvts/ivad115)
Supplement: ivad115_Supplementary_Data [file ivad115_supplementary_data.zip › INDURE 1Y_20230315 Supplementary Table.docx]

**Supplementary Table 1**: Quality of life

|  | **Baseline** | **3-6 months** | | **1 Year** | |
| --- | --- | --- | --- | --- | --- |
|  | **(N=397)** | **(N=394)** | **(N=378)** | **(N=380)** | **(N=365)** |
|  | Mean (SD) | Mean (SD) | p-value* (vs. Baseline) | Mean (SD) | p-value* (vs. Baseline) |
| **SF-12v2** |  |  |  |  |  |
| Physical functioning | 40.8 (11.9) | 48.7 (10.1) | <0.001 | 49.0 (9.8) | <0.001 |
| Role physical | 40.0 (11.8) | 46.5 (10.6) | <0.001 | 48.4 (10.4) | <0.001 |
| Bodily pain | 46.3 (12.0) | 49.7 (10.8) | <0.001 | 51.1 (10.2) | <0.001 |
| General health | 40.4 (11.2) | 47.2 (10.9) | <0.001 | 47.8 (10.2) | <0.001 |
| Vitality | 45.8 (11.7) | 51.4 (10.5) | <0.001 | 51.5 (11.5) | <0.001 |
| Social functioning | 44.4 (11.7) | 48.8 (10.3) | <0.001 | 49.5 (9.9) | <0.001 |
| Role emotional | 40.6 (13.2) | 46.2 (11.9) | <0.001 | 47.1 (11.4) | <0.001 |
| Mental health | 46.6 (11.1) | 51.7 (10.3) | <0.001 | 51.0 (10.5) | <0.001 |
| **Physical component summary** | 41.5 (10.5) | 47.7 (9.7) | <0.001 | 49.2 (9.5) | <0.001 |
| **Mental component summary** | 45.6 (11.2) | 50.0 (10.5) | <0.001 | 49.9 (10.6) | <0.001 |
| **KCCQ** | **(N=401)** | **(N=399)** | **(N=384)** | **(N=385)** | **(N=371)** |
| Physical limitations | 75.5 (23.1) | 89.4 (15.7) | <0.001 | 89.6 (17.5) | <0.001 |
| Symptom stability | 43.8 (18.4) | 57.9 (19.1) | <0.001 | 54.2 (16.0) | <0.001 |
| Symptom burden | 74.5 (24.1) | 87.8 (18.0) | <0.001 | 89.3 (17.8) | <0.001 |
| Symptom frequency | 74.6 (22.6) | 87.4 (17.4) | <0.001 | 88.6 (17.4) | <0.001 |
| Self-efficacy | 66.5 (27.8) | 81.3 (21.4) | <0.001 | 82.5 (21.0) | <0.001 |
| Quality of life | 49.9 (26.7) | 80.0 (22.9) | <0.001 | 83.1 (22.5) | <0.001 |
| Social limitations | 64.2 (29.6) | 83.9 (22.8) | <0.001 | 87.5 (21.0) | <0.001 |
| **Total symptom score** | 74.6 (22.6) | 87.6 (17.2) | <0.001 | 90.0 (17.1) | <0.001 |
| **Overall summary score** | 66.1 (22.7) | 85.2 (17.5) | <0.001 | 87.1 (18.0) | <0.001 |
| **Clinical summary score** | 75.1 (21.2) | 88.4 (15.6) | <0.001 | 89.2 (16.4) | <0.001 |

SD=standard deviation

*Based on paired cases
